# Pyruvate kinase M2 modulates Japanese encephalitis virus replication in neuronal cells

**DOI:** 10.1099/jgv.0.002140

**Published:** 2025-09-22

**Authors:** Vijay Singh Bohara, Atharva Deshmukh, Sachin Kumar

**Affiliations:** 1 Department of Biosciences and Bioengineering, Indian Institute of Technology Guwahati, Guwahati, India

**Keywords:** Japanese encephalitis virus, non-structural protein, pathogenesis, pyruvate kinase M2, replication

## Abstract

Japanese encephalitis is a neuroinflammatory condition caused by the Japanese encephalitis virus (JEV). Pyruvate kinase muscle isozyme M2 (PKM2) is a key modulator of glucose metabolism. The role of PKM2 in the autoimmune response and inflammation is now increasingly being acknowledged. However, its role in modulating virus replication has not been explored. In the current study, we have explored the role of PKM2 in JEV replication. Our results show that endogenous PKM2 expression is significantly upregulated in JEV-infected mouse neuroblastoma cells. Moreover, overexpression and knockdown studies substantiate the negative effect of PKM2 on JEV replication. Additionally, JEV infection induced signal transducers and activators of transcription 3 (STAT3) activation in the infected neuronal cells. Overexpression of PKM2 enhanced STAT3 activation, while its downregulation reduced STAT3 activation in the JEV-infected neuronal cells. The results suggested that the overexpression of PKM2 exhibited elevated levels of TNF-*α* and IL-1*β*, whereas the downregulation of PKM2 decreased their expression. The *in silico* studies revealed the potential interaction between PKM2 and non-structural protein 1 (NS1), which was subsequently validated *in vitro* by co-immunoprecipitation assay. The microscopic studies also unveiled the cellular co-localization of PKM2 and NS1 in the endoplasmic reticulum of infected cells. Altogether, these findings indicate that PKM2 negatively regulates JEV replication by inducing the expression of proinflammatory cytokines such as TNF-*α* and IL-1*β*. The study also establishes PKM2 as a binding partner of the NS1 protein. Thus, the study paves the path towards understanding the multifaceted role of PKM2 in JEV pathology.

## Importance

Japanese encephalitis is a major threat to public health not only because it causes many deaths but also because of its permanent neuropsychiatric sequelae, such as seizures, paralysis, motor dysfunction and cognitive impairment in children. Out of all non-structural proteins of Japanese encephalitis virus (JEV), non-structural protein 1 (NS1) is highly immunogenic. A wide range of possible interactive partners has been identified for the NS1, many of which have been linked to immune evasion and regulating viral replication. In the current study, we have described a novel host cell factor, pyruvate kinase isoform M2, regulating JEV replication, perhaps by modulating the expression of proinflammatory cytokines such as TNF-*α* and IL-1*β*. Considering PKM2’s central role in regulating the host cell metabolism, our findings suggest its role in JEV replication. Moreover, it paves the way for further research into the other novel transcription factors to elucidate the intricate relationship between JEV infection and the host’s immune response.

## Data Availability

The datasets generated in the study are available upon appropriate request from the corresponding author.

## Introduction

Japanese encephalitis is a serious vector-borne disease resulting in permanent neurological sequelae. The Japanese encephalitis virus (JEV) is the causative agent of Japanese encephalitis [1]. It has a widespread distribution across the world but is found more predominantly in South Asia, Southeast Asia and Western Pacific countries. The natural hosts of JEV are pigs and birds (mainly arid birds), which act as amplifying hosts and maintain their life cycle in nature, while humans and horses are dead-end hosts for JEV [2]. JEV is transmitted mainly through the bite of *Culex* mosquitoes [3].

JEV is classified as a member of the genus *Orthoflavivirus* under the family *Flaviviridae* [2]. The positive-sense 10.7 kb ssRNA genome of the JEV encodes a single polyprotein that is processed into three structural proteins (capsid, pre-membrane and envelope) and seven non-structural (NS) proteins (NS1, NS2A/B, NS3, NS4A/B and NS5) by viral and host proteases [4]. The replication of JEV genomic RNA occurs in the viral ‘replication complex’ that relates to membranes produced from the endoplasmic reticulum (ER) [5]. The replication process is facilitated by JEV non-structural proteins, in conjunction with various host factors [6,9]. Host factors employ diverse strategies to support or impede virus replication. Therefore, it is critical to elucidate the host factors that influence the replication cycle of JEV and investigate the underlying molecular mechanism.

NS1 protein is a highly conserved protein with a molecular weight ranging from 46 to 55 kDa, depending on the extent of glycosylation [10]. It exists in three forms: monomeric, dimeric and hexameric. The hydrophobic dimeric form is membrane-associated and is transported to viral replication sites to participate in viral replication. Dimeric NS1 is further processed into hexameric NS1, which is secreted out from the cell [11]. It has been proposed to provide a structural role, potentially contributing to the attachment of the replication complex to the membrane, alongside transmembrane replicase components [12]. Additionally, it has been found that the NS1 protein interacts with several host proteins. It has been shown that SLC25A12 enhances the type I IFN response by interacting with NS1 to suppress JEV replication [13]. Furthermore, NS1 and NS1′ proteins promote JEV replication by binding to CDK1, which translocates it from the nucleus to the cytoplasm, where it phosphorylates vimentin and remodels its network [14]. NS5 is the largest protein, with RNA-dependent RNA polymerase activity on its N-terminal motifs, while methyltransferase activity is at the C-terminal end. It also acts as an IFN antagonist [15]. NS3 acts as a viral helicase [16]. NS2A functions as a membrane-associated platform that transports viral RNA and both structural and non-structural viral proteins to the site of virion assembly. The NS2B binds with NS3, resulting in a functional protease complex that cleaves viral polyprotein into functional proteins. NS4A forms membrane curvature, resulting in the formation of a scaffold for the replication complex. NS4B impairs the host’s immune response by inhibiting IFN signalling [17].

RNA viruses have been shown to modulate key glycolytic enzymes such as hexokinase, phosphofructokinase, pyruvate kinase and lactate dehydrogenase (LDH) [18,22]. Some of these glycolytic enzymes also have non-metabolic roles, especially in regulating innate immune responses. Hexokinase was found to be upregulated in inflammatory diseases [23,24]. Its inhibition by 2-deoxy-d-glucose (2-DG) has been shown to inhibit the expression of proinflammatory cytokines [25]. In the case of Zika virus infection, it has been reported that treatment with 2-DG resulted in enhanced innate immune responses through the activation of AMP-activated protein kinase, suppressing its replication [19]. Furthermore, porcine reproductive and respiratory syndrome virus (PRRSV) infection was also found to induce glycolysis by upregulating hexokinase and LDH in the infected cells [21]. The LDH converts pyruvate into lactate, which in turn reduces RIG-I, MDA5 and MAVS-induced IFN-*β* promoter activity, thereby suppressing innate immune responses in PRRSV-infected cells [21]. LDH and aldolase were also found to modulate JEV replication [26,27]. Pyruvate kinase muscle isozyme M2 (PKM2) is one of the key glycolytic enzymes which has been well explored in terms of its roles in cancer. However, its role in virus replication remains unclear. While some studies do suggest its involvement in the replication of viruses [28,31], there is currently no evidence suggesting its role in JEV replication.

PKM2 is a rate-limiting glycolytic enzyme, essential for tumour metabolism and development [32]. PKM2 catalyses the conversion of phosphoenol pyruvate (PEP) to pyruvate, the last ATP-generating step of glycolysis [33]. PKM2 also plays a pivotal role in multiple cellular pathways, encompassing aerobic glycolysis, intranuclear signal transmission, protein synthesis, inflammation and apoptosis [34]. PKM2 enhances the expression of genes by activating hypoxia-inducible factor-1*α* (HIF-1*α*), *β*-catenin (*β*-cat), insulin, signal transducers and activators of transcription 3 (STAT3) and other transcription factors that stimulate cell growth and proliferation [35,37]. PKM2 also serves as a critical regulator in the apoptotic signalling pathways of several cancer types [38,40]. B-cell lymphoma 2, a prominent anti-apoptotic protein and very well characterized for its anti-apoptotic properties, is a target of PKM2, both directly and indirectly [38]. As part of the innate immune response, PKM2 stimulates the production of inflammatory cytokines such as IL-1*β* and TNF-*α* [41]. The PKM2 also functions as a protein kinase, facilitating the phosphorylation of STAT3, leading to the production of IL-6 and IL-1*β* to initiate the inflammatory response [42]. PKM2 activates and engages HIF-1*α* to regulate the release of high mobility group box-1, an efficient proinflammatory cytokine that is released from activated macrophages [43,44]. Recent studies have shown the role of PKM2 expression in autoimmune and inflammatory responses as well [45,47].

PKM2 is one of the key glycolytic enzymes which performs several non-glycolytic tasks with far-reaching consequences, the extent of which has to be fully deciphered [48]. PKM2 has been associated with several RNA virus infections. The expression of PKM2 was enhanced in mouse lung tissues infected with the influenza virus H1N1 [49]. PKM2 has also been shown to be elevated in individuals affected with severe coronavirus disease 2019 [50]. Phosphorylation of PKM2 was also found to be enhanced upon dengue virus infection in U937 cells [51]. Phosphorylation inhibits its catalytic activity and promotes its translocation to the nucleus [51,52]. Studies in classical swine fever virus and hepatitis C virus have also shown PKM2 involvement in virus replication [53,54]. Collectively, these studies suggest the potential involvement of PKM2 in RNA virus pathogenesis. However, there is no report about its role in JEV replication.

The present study focuses on investigating the potential role of PKM2 in JEV replication. This was accomplished by examining the effects of overexpression of PKM2 and silencing of endogenous PKM2 on JEV replication in mouse neuroblastoma cells. *In-silico* and microscopic analyses were also performed to investigate the potential interaction between PKM2 and NS1. This was further confirmed *in vitro* using the co-immunoprecipitation (Co-IP) technique. We explored possible molecular mechanisms by which PKM2 could regulate the JEV infection in the infected cells.

## Methods

### Cells and virus

The mouse neuroblastoma Neuro-2a and the baby hamster kidney BHK-21 were obtained from the National Centre for Cell Sciences, Pune, India. The cells were cultured in Dulbecco’s modified Eagle medium (DMEM) with 10% FBS and 1% antibiotic-antimycotic (GIBCO) cocktail at 37 °C in a humidified incubator in 5% CO2. The JEV strain SA14-14-2 (GenBank accession number JN604986) used in this study was procured from the Department of Health and Family Welfare, Government of Assam, India. The JEV stock preparation and its titration by plaque assay were performed in BHK-21 cells. For stock preparation, BHK-21 cells at 80% confluency were infected with JEV at 0.1 m.o.i. Following a period of 2 h during which the JEV was allowed to adsorb to the cells, the infection media was subsequently substituted with DMEM media supplemented with 2% FBS. Cells were lysed in successive freeze-thaw cycles 72 h post-infection. Cell debris was removed by centrifuging at 1,500 ***g*** for 5 min, and the clear supernatant was collected and stored at −80 °C. Furthermore, the presence of JEV was confirmed by plaque assay.

### Plasmids and antibodies

The following plasmids were used in the current study: pEGFP-PKM2 (Addgene #64698) and pEGFP (Addgene #165830). The antibodies used are as follows: anti-NS1 (GeneTex, USA), anti-PKM2 (Cell Signaling Technology, USA), anti-GAPDH (Cell Signaling Technology, USA), anti-GFP (Bio Bharati, India), goat anti-rabbit (Invitrogen, USA) and anti-mouse (Cell Signaling Technology, USA) conjugated with HRP.

### Virus infection and quantification

Neuro-2a cells were seeded in a six-well plate at a density of 0.6×10^6^ cells well^−1^ and infected with JEV at 0.1 m.o.i. The virus was allowed to adsorb for 2 h with intermittent shaking. Thereafter, the infection media were replaced with fresh DMEM (2 ml well^−1^) supplemented with 2% FBS, and the plate was placed in 5% CO_2_. Cells and supernatants from JEV-infected cells were collected at different time points post-infection. Collected cells were used for expression analysis of the endogenous PKM2 and JEV NS1 gene and protein using quantitative real-time PCR and immunoblotting. The collected supernatant was used for virus quantification using the plaque assay.

### Gene expression analysis using real-time PCR

RNA lysate was prepared at different time points post-infection using RNAiso Plus reagent (TaKaRa, Japan). Total RNA was extracted using the phenol-chloroform extraction method, and 1 µg of total RNA was reverse transcribed into cDNA using a high-capacity cDNA reverse transcription kit (Thermo Fisher Scientific, USA). The quantitative real-time PCR was performed using PowerUp SYBR Green Master Mix (Applied Biosystems, USA). The fold change in mRNA level in infected versus mock-infected samples was calculated using the 2^(−ΔΔ*Ct*)^ method with glyceraldehyde-3-phosphate dehydrogenase (GAPDH) as an internal control for normalization.

### Immunoblotting

Cells were lysed in RIPA (radioimmunoprecipitation assay buffer), and proteins were separated on 12% SDS gel. Separated proteins from the gel were transferred to a 0.4 µm nitrocellulose membrane. The membrane was blocked with 5% skimmed milk prepared in 1X TBST (Tris-Buffered Saline with Tween-20) for 2 h. Following blocking, primary antibody dilution was prepared in 2% BSA and incubated at 4 ˚C overnight. After incubation, the membrane was washed with PBS and thereafter incubated with HRP-conjugated secondary antibody for 1 h at room temperature and detected using ECL (Enhanced Chemiluminescence) reagent (BioRad, USA) under chemiluminescence. Quantitative analysis was performed for each biological replicate using ImageJ software.

### Overexpression and knockdown studies

The plasmid used in this study, pEGFP-PKM2 (Catalogue No. #64698), was a generous gift from the Axel Ullrich lab [55]. For overexpression experiments, Neuro-2a cells were cultured on six-well plates and then transfected with a total of 2 µg of an expression plasmid using Lipofectamine 2000 (Invitrogen, USA) as per the instructions provided by the manufacturer.

The knockdown of endogenous PKM2 was achieved by co-transfecting two siRNAs at a total concentration of 50 pmol. The following sense strand sequences of siRNA were used: GAUGUCGACCUUCGUGUAA[dT] and UCCUAUCAUUGCCGUGACU[dT][dT] [56]. Lipofectamine RNAiMAX (Invitrogen, USA) was used for transfection studies as per the manufacturer’s protocol, and siRNA universal negative control (SIC001, Sigma-Aldrich, Germany).

### Disuccinimidyl suberate (DSS) cross-linking of PKM2

Neuro-2a cells were infected with JEV, and 48 h post-infection, cells were washed with ice-cold PBS three times and treated with 500 µM of DSS crosslinker (Merck, Catalogue No. S1885) for 30 min at 37 ˚C. The cross-linking reaction was quenched by treating cells with 10 mM Tris/HCl (pH 7.5) for 15 min. Cells were then lysed with RIPA buffer, followed by western blotting.

### Nuclear and cytoplasmic fractionation

The Neuro-2a cells were cultured in a six-well plate and infected with 0.1 m.o.i. JEV. At 48 h post-JEV infection, cells were collected and washed with PBS twice. The cells were lysed in lysis buffer containing PBS, 1 mM dithiothreitol, protease inhibitor cocktail (TaKaRa) and 1% NP 40 for 10 min at room temperature. The lysate was centrifuged at 1,000 ***g*** at 4 ˚C for 10 min. The supernatant containing the cytoplasmic fraction was collected in separate tubes. The nuclear pellet was washed with PBS twice and lysed in 40 µl of 1% SDS. The collected fractions were then subjected to SDS-PAGE followed by western blotting. GAPDH and H3 histone served as internal controls for fractionation. ** **

### Structure modelling of JEV NS1 and mouse PKM2 protein

The amino acid sequences of JEV NS1 and mouse PKM2 proteins were retrieved from GenBank accession numbers QCZ42158 and NP_001365797, respectively. The three-dimensional structures of the proteins were predicted and generated by Iterative Threading ASSEmbly Refinement protein structure and function prediction software, as experimentally validated complete structures were not available for the above proteins. The tool constructs 3D structure models by threading the query protein sequence through a library of known structures in the Protein Data Bank (PDB), followed by iterative template-based fragment assembly simulation [57,59]. Furthermore, the predicted models were subsequently subjected to energy minimization using YASARA software to enhance the structural stability and reduce steric clashes. Thereafter, the models were validated by the Ramachandran plot using PROCHECK, which assesses its stereochemical stability based on dihedral angle distributions [60]. All three-dimensional structures were visualized using PyMOL.

### Molecular docking study

To study the possible interaction between the predicted structure of JEV NS1 and mouse PKM2 protein, molecular docking was performed using ClusPro 2.0 protein–protein docking software. ClusPro 2.0 generates multiple docking conformations (clusters) based on rigid-body docking, followed by energy-based filtering and clustering to identify the most probable binding models [61]. The top-ranked docked complexes from ClusPro 2.0 were further analysed for binding affinity using the HawkDock server scoring function, which is based on molecular mechanics/generalized born surface area (MM/GBSA) free energy decomposition [62,63]. The MM/GBSA method provides a more rigorous estimation of binding free energy for protein–protein complexes, where lower binding energy values indicate higher stability of the complex [64,65]. Finally, the PDB sum generator tool was used to get insights into the amino acid residues involved in binding over the molecular interface spanning both proteins [66].

### Molecular dynamics (MD) simulations

To study the conformational stability, the docked protein–protein complex was subjected to all-atom MD simulations using the GROMACS v2020.1 software [67,68]. The system consisted of the protein–protein complex in a solvated dodecahedron box with a minimum distance of 1.2 nm from the boundary. The system was solvated with the TIP3P water model and subsequently neutralized by adding countercations (Na^+^) or anions (Cl^−^). The solvated system was then energy minimized using the steepest descent algorithm of 50,000 steps and further equilibrated for constant number, pressure and temperature and constant number, volume and temperature at 300 K, 1 bar pressure and 100 ps in order to optimize the orientation and system density. The final equilibrated system was used as a starting conformation for the simulation run of 50 ns with a 2 fs time step. Long-range electrostatic interactions were defined by the Particle Mesh Ewald approach, and the cut-offs for Coulomb and van der Waals interactions were set to 1.2 nm. Finally, the output trajectory was obtained, and the estimation of root mean square deviation (RMSD), root mean square fluctuation (RMSF), solvent accessible surface area (SASA) and radius of gyration (Rg) was performed using GROMACS packages. The graphs were analysed and plotted using GraphPad Prism software.

### Immunofluorescence

Neuro-2a cells were cultured on coverslips and subsequently transfected with pEGFP-PKM2. The cells were infected with JEV 24 h post-transfection, and 48 h post-infection, cells were fixed with 4% formaldehyde in PBS for 15 min at room temperature. Following fixation, cells were washed with PBS at least three times and permeabilized with 0.3% Triton X-100 in PBS. Subsequently, the fixed cells were rinsed using 1X PBS and then incubated in a blocking buffer (5% BSA). For JEV protein detection, cells were incubated with a 1/100 dilution of mouse mAb against NS1 (GeneTex, USA), followed by incubation with a 1/500 dilution of goat Alexa Fluor 594 (Invitrogen, USA) anti-mouse IgG secondary antibody. After the final wash with PBS, cells were stained with DAPI, and slides were mounted using Prolong Gold Antifade Reagent (Invitrogen, USA) and examined under a 63X objective using the confocal microscope (Zeiss LSM 880, Germany).

### Co-IP assay

Capturem Co-IP kit was procured from TaKaRa (Japan). The Neuro-2a cells were transfected with pEGFP-PKM2, and the cells were infected with JEV 24 h post-transfection. Infected cells were harvested and lysed after 48 h in the 200 µl lysis buffer per 1×10^6^ cells. An appropriate amount of the protease inhibitor cocktail was added to the lysis buffer to yield a 1X final concentration. The collected lysate was centrifuged at 17,000 ***g*** for 10 min at 4 ˚C. The clear supernatant was collected and incubated with the recommended amount of anti-GFP (Bio Bharati, India) and anti-NS1 (GeneTex, USA) antibodies for 3 h at 4 ˚C. Pre-incubated samples were loaded onto the Capturem protein A columns (TaKaRa) and centrifuged at 1,000 ***g*** for 1 min at room temperature. Flowthrough was discarded, and spin columns were washed with wash buffer (provided in the kit). Bound samples were eluted from spin columns in elution buffer by centrifugation at 1,000 ***g*** for 1 min at room temperature and analysed by Western blot.

### Statistical analysis

All the data were statistically validated using GraphPad Prism software, and results were shown as mean±sd. All the experiments were reproduced at least three independent times. The significance level among different groups was represented as *, ** and ***, where * is for *P*<0.05, ** for *P*<0.01 and *** for *P*<0.001. *P*<0.05 was considered significant.

## Results

### JEV infection induces upregulation of PKM2 expression in Neuro-2a cells

**Fig. 1.**
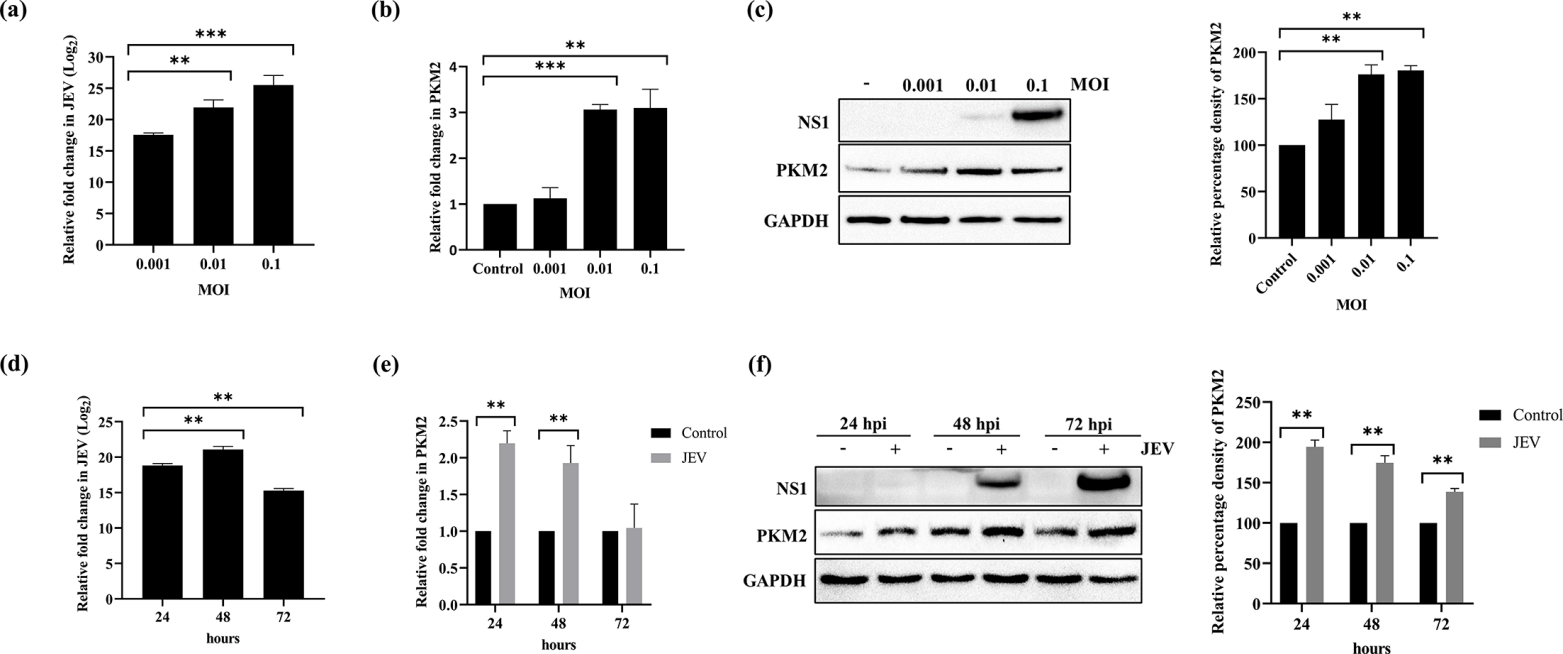
JEV infection leads to upregulation of PKM2. Real-time PCR analysis of JEV replication at different m.o.i. post-infection (**a**). Fold change in PKM2 mRNA at different m.o.i. post-infection (**b**). Immunoblot showing m.o.i.-dependent expression of PKM2 and NS1 at protein level (**c**). Time-dependent analysis of JEV replication (**d**). Time-dependent fold change in PKM2 mRNA expression post-infection (**e**). Immunoblot analysis of PKM2 and NS1 expression at different time points post-infection (**f**). GAPDH was used for the normalization of real-time PCR analysis. Values are shown as mean fold change±sd validated with one-way ANOVA statistical test. The significance level among different groups was represented as *, ** and ***, where * is for *P*<0.05, ** for *P*<0.01 and *** for *P*<0.001. *P*<0.05 was considered significant.

The expression of PKM2 was investigated in Neuro-2a cells following JEV infection with different m.o.i. Quantitative analysis using real-time PCR at various m.o.i. showed a gradual increase in JEV replication in infected cells (Fig. 1a). PKM2 mRNA also showed an increase in expression compared to the control with the increase in m.o.i., except at 0.001 (Fig. 1b). However, the maximum increase in expression was at 0.1 m.o.i. At the protein level, there was a significant increase in PKM2 expression at both 0.01 and 0.1 m.o.i. compared to the uninfected control. However, there was no change in PKM2 expression at 0.001 m.o.i. compared to the uninfected control (Fig. 1c). Furthermore, the time-dependent experiment was performed to investigate the progression of JEV replication over time (Fig. 1d). The mRNA analysis revealed an upregulation of PKM2 expression at both 24 and 48 h post-infection. There was no significant difference in the PKM2 mRNA compared to the control at 72 h post-infection (Fig. 1e). At the protein level, there was a significant increase in PKM2 expression in infected cells compared to the uninfected control (Fig. 1f). Thus, JEV replication induces PKM2 expression in the infected cells.

### PKM2 expression is responsible for inhibiting JEV replication

**Fig. 2.**
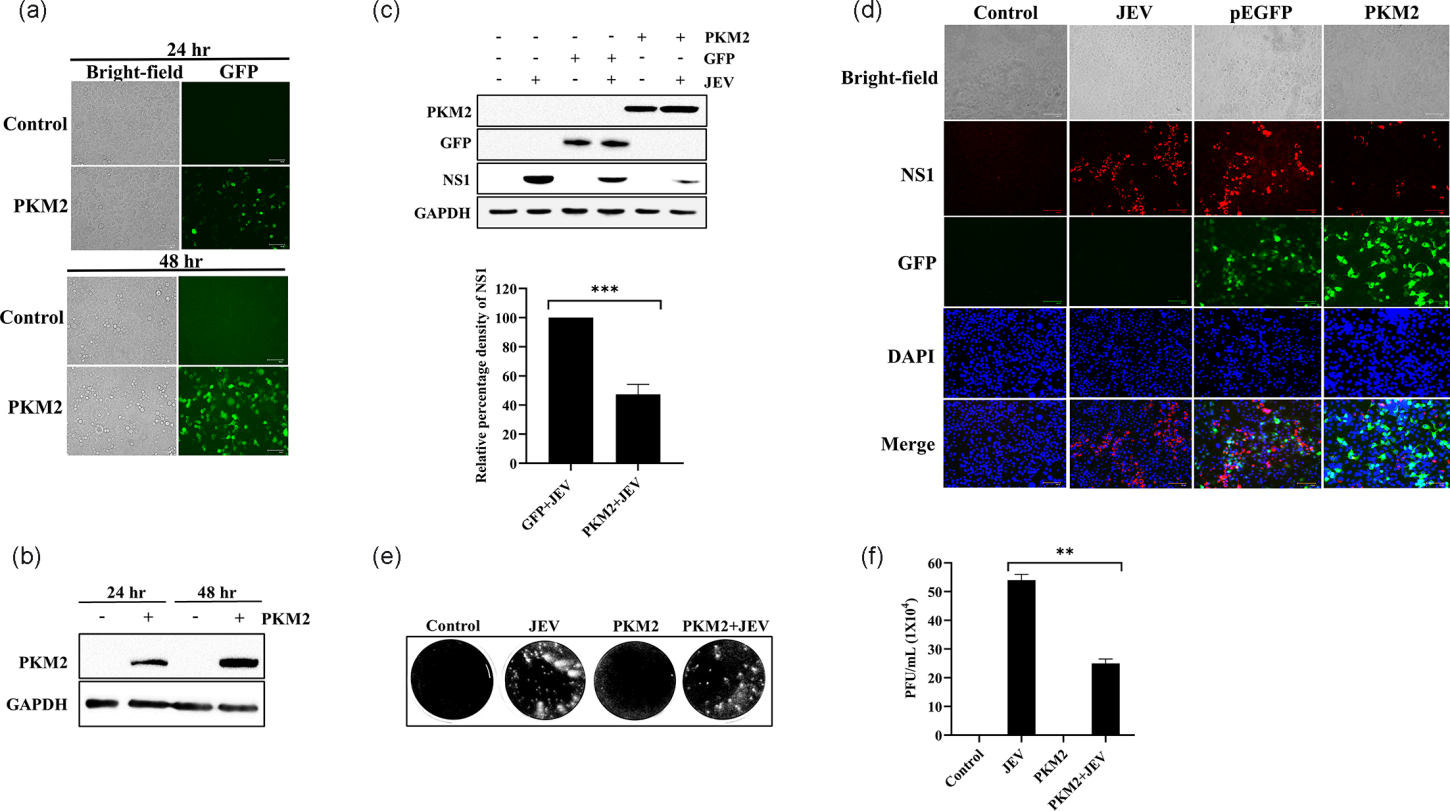
PKM2 overexpression inhibited JEV replication. The image shows the expression of exogenous PKM2 24 and 48 h post-transfection (**a**). Immunoblot analysis of time-dependent expression of exogenous PKM2 post-transfection (**b**). Immunoblot analysis of NS1 expression upon overexpression of exogenous PKM2 in infected cells (**c**). Immunofluorescence pictures of cells with exogenous PKM2 expression and JEV infection. Immunofluorescence labelling of JEV; red fluorescence indicates JEV-infected cells; blue fluorescence indicates DAPI-labelled nuclei; green fluorescence represents PKM2 expression (**d**). Picture showing the reduction in extracellular viral titre in the form of plaques (**e**). Graph representing virus titre level measured in p.f.u. ml^−1^ (**f**). Values are shown as mean±sd validated with one-way ANOVA statistical test. The significance level among different groups was represented as *, ** and ***, where * is for *P*<0.05, ** for *P*<0.01 and *** for *P*<0.001. *P*<0.05 was considered significant.

To investigate the function of PKM2 in the context of JEV infection, Neuro-2a cells were transfected with pEGFP-PKM2 plasmid (Fig. 2a). Exogenous PKM2 expression was confirmed at both 24 and 48 h post-transfection (Fig. 2b). Next, the cells were infected with JEV at 0.1 m.o.i., 24 h post-transfection, and protein lysate was prepared 48 h post-infection. The western blot analysis revealed around 52% reduction in NS1 expression in cells with overexpressed exogenous PKM2 compared to GFP control (Fig. 2c). This was further validated in an immunofluorescence experiment that showed a reduction (around 58%) in the number of cells expressing NS1 when exogenous PKM2 was overexpressed compared to GFP control (Fig. 2d). Using the standard plaque assay, the virus titre in the supernatant collected from the infected cells was also quantified (Fig. 2e). It was observed that there was ~54% reduction in JEV titre in cells overexpressing PKM2 compared to the control (Fig. 2f). In conclusion, the expression of exogenous PKM2 has a negative impact on JEV replication.

### PKM2 downregulation enhanced JEV replication

**Fig. 3.**
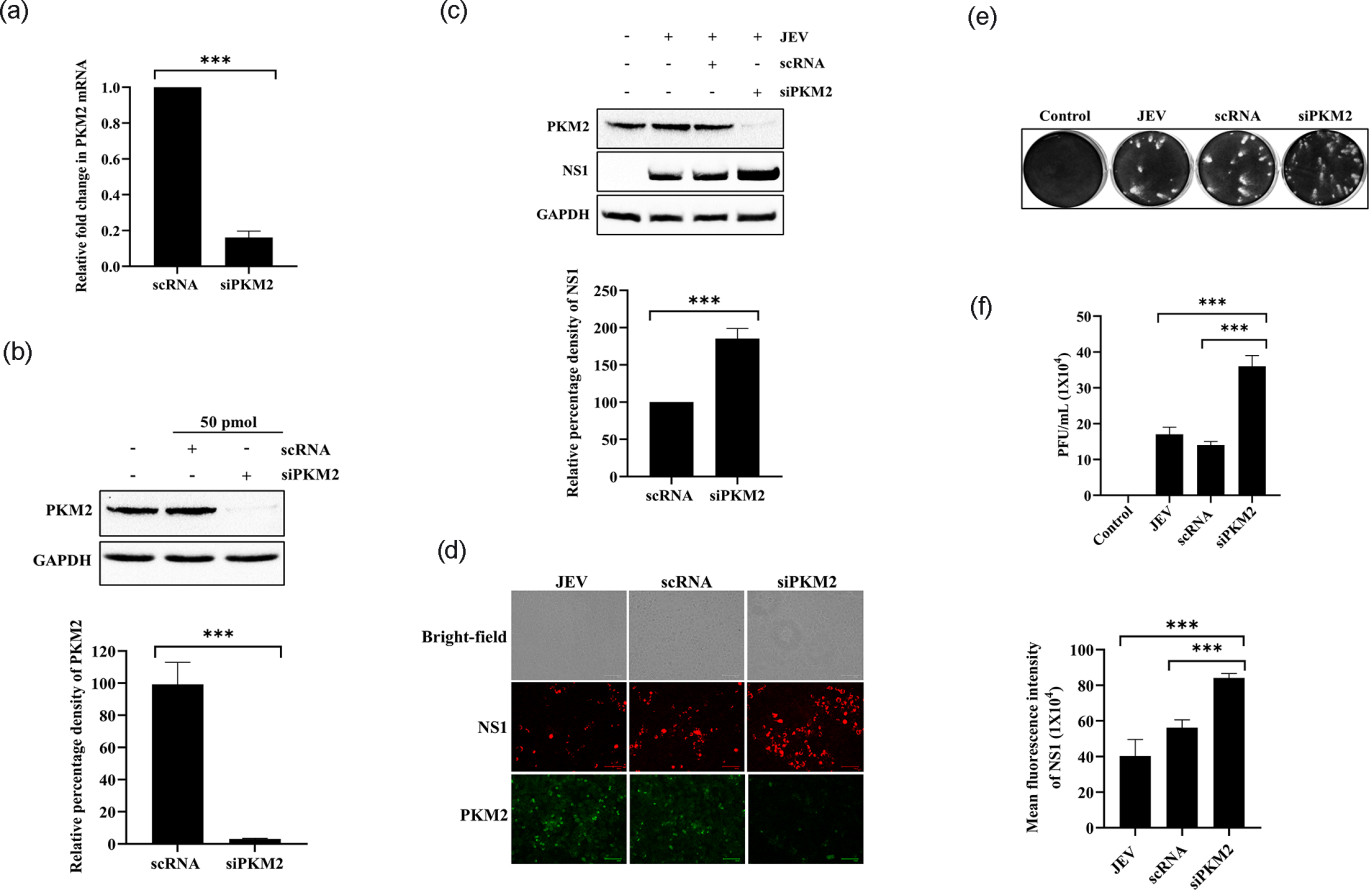
PKM2 knockdown enhanced JEV replication. Graph showing the reduction in PKM2 mRNA level upon treatment with PKM2 siRNA (**a**). Immunoblot showing a reduction in PKM2 protein level upon treatment with PKM2 siRNA (**b**). Immunoblot analysis of NS1 expression upon knockdown of PKM2 in infected cells (**c**). Immunofluorescence images showing enhanced JEV infection upon treatment with PKM2 siRNA. Red fluorescence images indicate JEV-infected cells, while green fluorescence indicates endogenous PKM2 expression (**d**). Plaque images showing the reduction in extracellular viral titre (**e**). Graph representing virus titre level in p.f.u. ml^−1^ (**f**). Values are shown as mean±sd validated with one-way ANOVA statistical test. The significance level among different groups was represented as *, ** and ***, where * is for *P*<0.05, ** for *P*<0.01 and *** for *P*<0.001. *P*<0.05 was considered significant.

Endogenous PKM2 was knocked down via transfection of a cocktail consisting of two PKM2-specific siRNAs. At the mRNA level, around 84% reduction in PKM2 expression was observed compared to scRNA-transfected cells (Fig. 3a). At the protein level, the transfection of specific siRNA resulted in ~99% reduction in PKM2 expression compared to scRNA-transfected cells (Fig. 3b). The western blot analysis demonstrated ~80% increase in NS1 expression upon PKM2 knockdown when compared to scRNA-transfected cells (Fig. 3c). Immunofluorescence results also showed an increase (around 62%) in the number of cells expressing NS1 upon PKM2 downregulation (Fig. 3d). Extracellular virus was titrated using a plaque assay (Fig. 3e). When compared to scRNA-transfected cells, supernatant from PKM2-silenced cells showed a 2.56-fold higher JEV titre, and compared to untransfected cells, titre was 2.1-fold higher (Fig. 3f). From these studies, we concluded that PKM2 silencing positively regulated JEV replication.

### Inhibition of PKM2 with metformin enhanced JEV infection

**Fig. 4.**
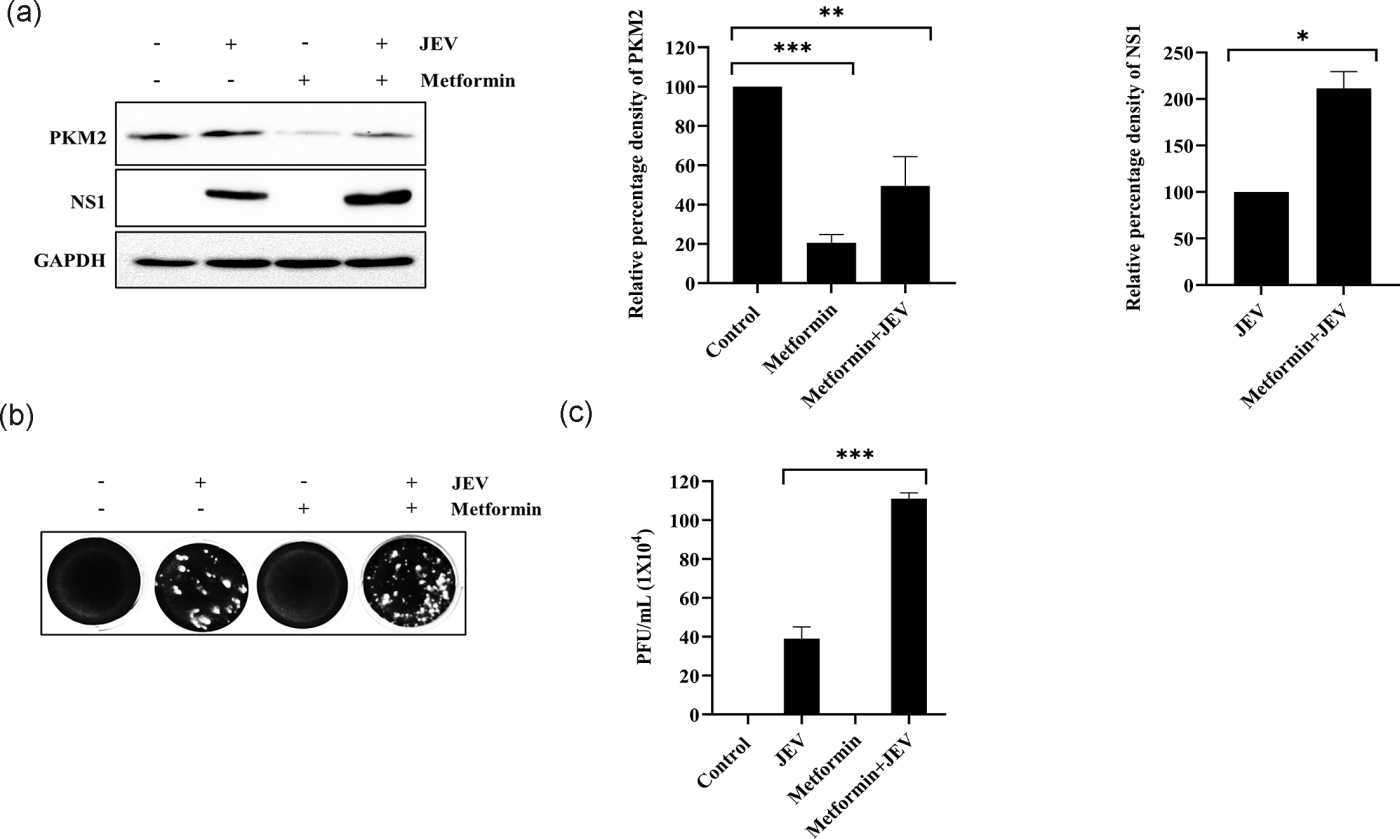
Inhibition of PKM2 with metformin enhanced JEV infection. Immunoblot indicates NS1 and PKM2 expression upon metformin treatment (**a**). Representative plaque image with enhanced JEV titre upon metformin treatment (**b**). Graph representing virus titre in p.f.u. ml^−1^ (**c**). Values represent mean±sd calculated from three independent experiments. Statistical analysis was performed using student’s t-test, considering *P*<0.05 as significant (* for *P*<0.05, ** for *P*<0.01 and *** for *P*<0.001).

JEV-infected cells were treated with 100 µM of metformin post-infection. At the protein level, ~79% decrease in endogenous PKM2 expression was observed, while in JEV-infected cells, ~50.5% decrease was observed compared to the untreated control (Fig. 4a). The amount of NS1 protein was also doubled (111% increase) in metformin-treated cells (Fig. 4a). Plaque assay also revealed about a 2.8-fold increase in virus titre in metformin-treated cells compared to untreated cells (Fig. 4b, c).

### PKM2 modulates JEV replication through STAT3 activation

**Fig. 5.**
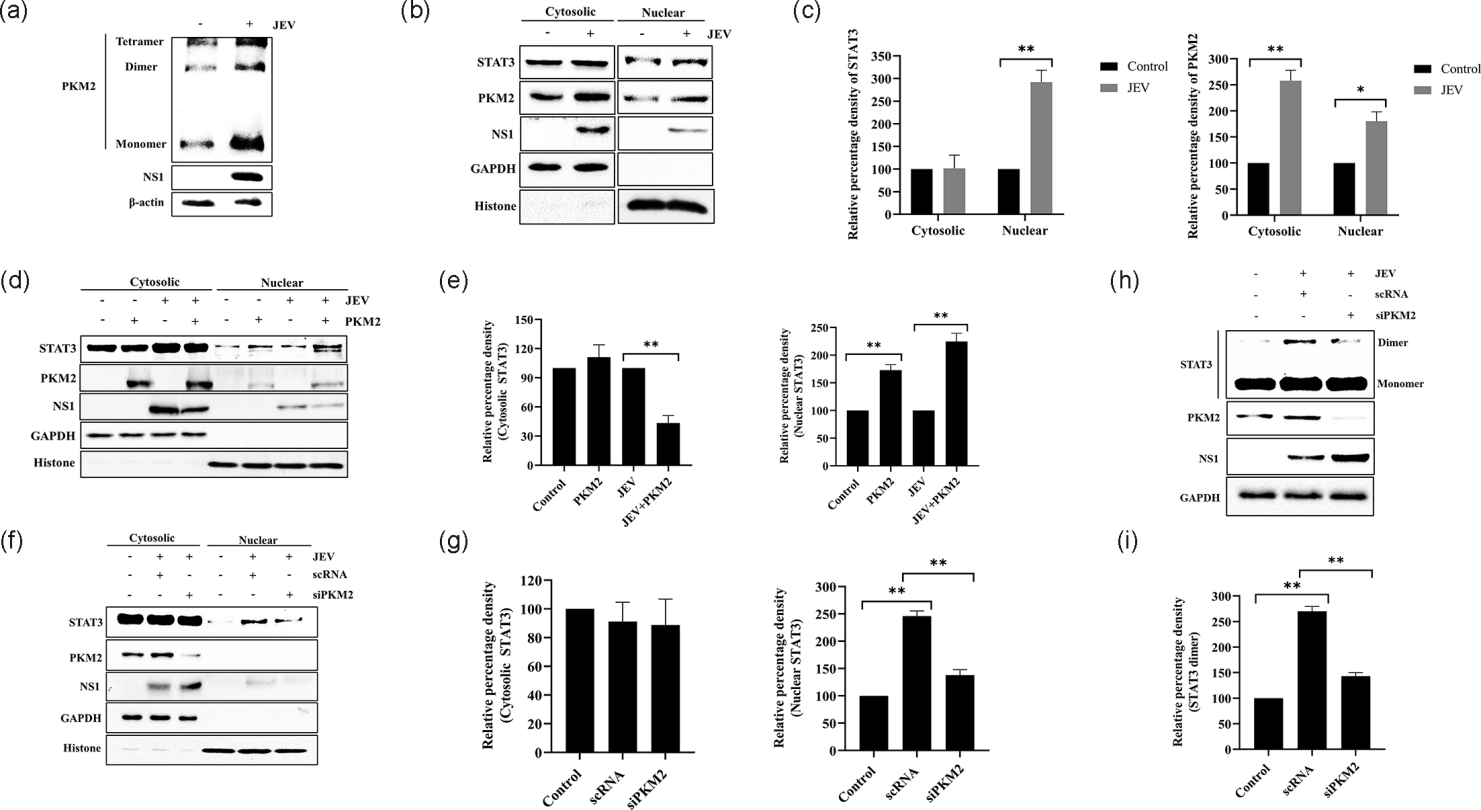
PKM2 modulates JEV replication through STAT3 activation. Immunoblot depicting different isoforms of PKM2 post-JEV infection (**a**). Western blot showing nuclear localization of STAT3 and PKM2 upon JEV infection (**b**) and graphs showing the quantitative analysis of western blot (**c**). Immunoblot showing nuclear translocation of STAT3 upon PKM2 overexpression in JEV-infected cells (**d**) and graphs showing the quantitative analysis of immunoblot (**e**). Immunoblot showing nuclear translocation of STAT3 upon PKM2 downregulation (**f**) and its quantitative analysis (**g**). Immunoblot representing dimeric STAT3 upon PKM2 knockdown in infected cells (**h**) and a graph showing its quantitative analysis (**i**). All the lysates were prepared 48 h post-JEV infection. Values represent mean±sd calculated from three independent experiments. Statistical analysis was performed using student’s t-test, considering *P*<0.05 as significant (* for *P*<0.05, ** for *P*<0.01 and *** for *P*<0.001).

To investigate the expression level of different multimeric forms of PKM2 post-infection, protein lysate collected from infected cells was cross-linked. The western blot revealed significantly enhanced expression of all forms (monomer, dimer and tetramer) of PKM2 post-infection (Fig. 5a). Nuclear and cytoplasmic fractions showed significantly enhanced translocation of STAT3 and PKM2 to the nucleus in JEV-infected cells compared to the uninfected control (Fig. 5b, c). To confirm the role of PKM2 in STAT3 activation post-JEV infection, overexpression and knockdown experiments were performed. Overexpression of PKM2 significantly enhanced STAT3 translocation to the nucleus post-JEV infection (Fig. 5d, e), while its downregulation substantially reduced STAT3 translocation to the nucleus in the infected cells (Fig. 5f, g). Downregulation of PKM2 also reduced dimeric STAT3 in JEV-infected cells (Fig. 5h, i). All these results show that JEV replication induces the nuclear translocation of STAT3 by upregulating PKM2 expression in the infected cells.

### PKM2 inhibits JEV replication by upregulating proinflammatory cytokines

**Fig. 6.**
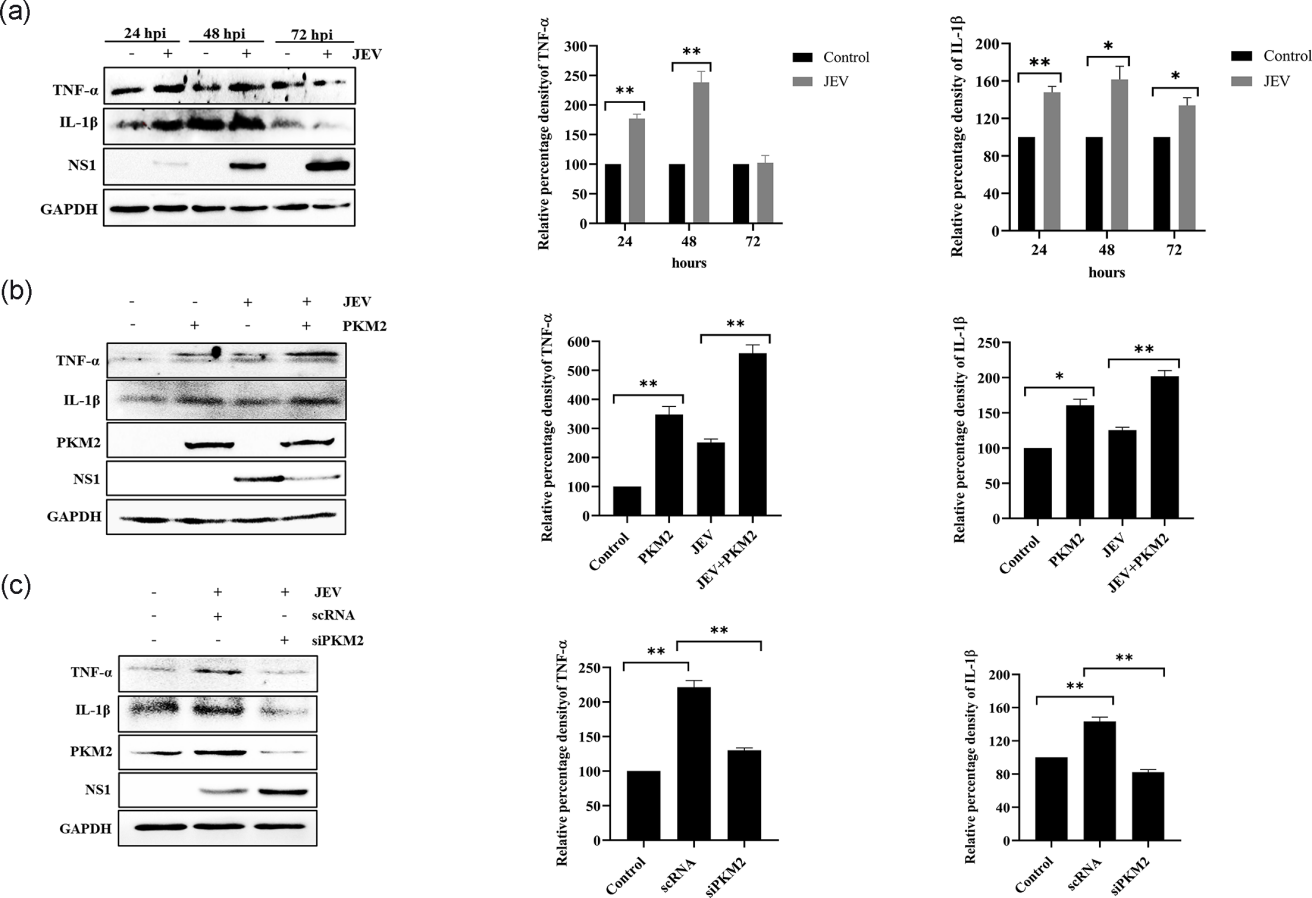
PKM2 inhibits JEV replication by upregulating proinflammatory cytokines. Western blot representing time-dependent analysis of TNF-*α* and IL-1*β* post-JEV infection (**a**). Immunoblot depicting effect of PKM2 overexpression on TNF-*α* and IL-1*β* (**b**). Immunoblot analysis of TNF-*α* and IL-1*β* expression upon PKM2 downregulation. For (**b**) and (**c**), lysates were prepared 48 h post-JEV infection. Values represent mean±sd calculated from three independent experiments. Statistical analysis was performed using student’s t-test, considering *P*<0.05 as significant (* for *P*<0.05, ** for *P*<0.01 and *** for *P*<0.001).

To investigate the expression of proinflammatory cytokines such as TNF-*α* and IL-1*β* post-JEV infection, a time-dependent experiment was performed. It was observed that expression of TNF-*α* and IL-1*β* significantly enhanced at early time points (24 and 48 h) post-infection (Fig. 6a). The overexpression of PKM2 significantly upregulated the levels of TNF-*α* and IL-1*β* (Fig. 6b), whereas the downregulation of PKM2 resulted in the significant reduction of these cytokines (Fig. 6c). This indicates that PKM2 suppresses JEV infection by inducing the expression of TNF-*α* and IL-1*β*.

### Interaction of PKM2 and NS1

**Fig. 7.**
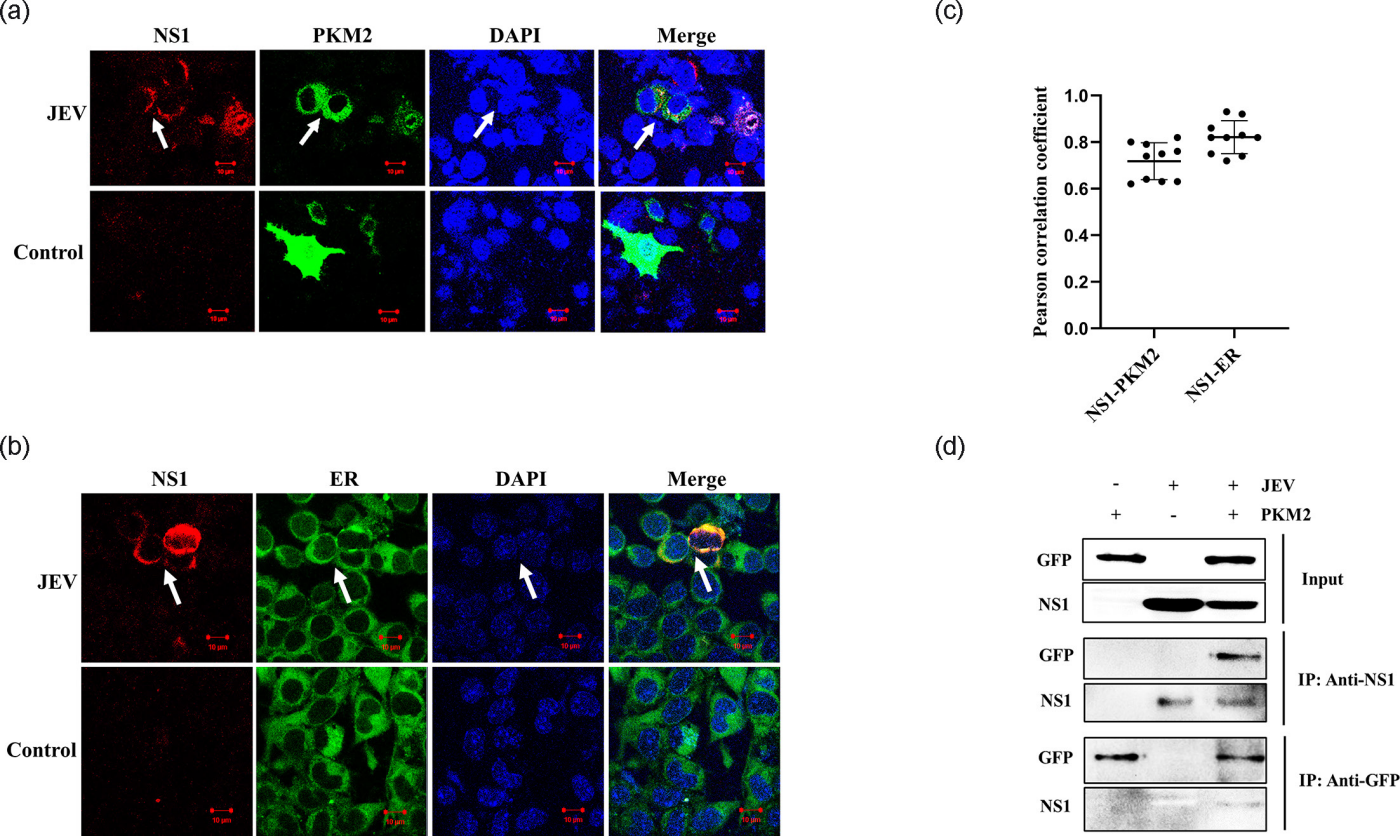
Interaction of PKM2 and NS1. Confocal images showing cellular co-localization of PKM2 with NS1 in JEV-infected cells (**a**). Representative images showing red fluorescence indicate NS1 expression, while green fluorescence indicates PKM2 expression. The merge panel shows both PKM2 and NS1 proteins. Cell nuclei were stained with DAPI (blue channel). Arrows indicate cells showing co-localization of PKM2 and NS1 (**a**). Confocal images showing localization of NS1 (red channel) and ER (green channel) in the JEV-infected cells (**b**). ER was stained with ER tracker dye, while the nucleus was stained with DAPI. Arrows indicate the localization of NS1 in the ER of the infected cells. The graph shows the average Pearson correlation coefficient of PKM2-NS1 and NS1-ER calculated using ten individual cells from three independent experiments (**c**). The interaction of PKM2 with the NS1 protein of JEV was examined by Co-IP assay. The cell lysates were immunoprecipitated by anti-NS1 and anti-GFP antibodies, and interactions were detected using immunoblot (**d**). Statistical analysis was performed using student’s t-test, considering *P*<0.05 as significant (* for *P*<0.05, ** for *P*<0.01 and *** for *P*<0.001).

To investigate the possible PKM2 and JEV NS1 interaction *in silico*. The three-dimensional structure of mouse PKM2 and JEV NS1 protein was modelled (Fig. S1, available in the online Supplementary Material). Docking between mouse PKM2 and JEV NS1 identified the most stable complex with the binding free energy of −150.85 kcal mol^−1^ (Fig. S2A). Ramachandran plot analysis showed 83.3% of residues in the most favourable regions, indicating decent stereochemical stability (Fig. S2B). Analysis of interacting residues revealed 33 residues from each partner (Fig. S2C), forming 6 salt bridges, 24 hydrogen bonds and 372 non-bonded contacts (Table S1). MD simulations were performed to test whether the PKM2-NS1 complex was stable over time in a simulated physiological environment. Several parameters, such as RMSD, RMSF, Rg and SASA, were analysed for PKM2, NS1 and PKM2-NS1 complex (Fig. S3). Overall, these parameters showed that the PKM2-NS1 complex was stable and rigid. Confocal microscopy was performed to further study the cellular localization of PKM2 and NS1. Cells were transfected with a PKM2-expressing plasmid and infected with JEV. Immunofluorescence results showed the cellular co-localization of PKM2 with NS1 protein (Fig. 7a). JEV NS1 protein was also found to localize in the ER of infected cells (Fig. 7b). PKM2 showed a strong correlation with NS1 (Pearson correlation coefficient 0.72), and NS1 showed a strong correlation with ER (Pearson correlation coefficient 0.82) in the infected cells (Fig. 7c). The Co-IP experiment was performed to further validate the potential interaction between PKM2 and NS1. PKM2 was detected after the pull-down with NS1, while NS1 was detected after the pull-down with GFP. This validated the interaction between PKM2 and NS1 protein (Fig. 7d). From these studies, we conclude that PKM2 and NS1 interact with each other, possibly in the ER of infected cells.

## Discussion

PKM2 is a glycolytic enzyme catalysing the rate-limiting, last step of glycolysis [32]. PKM2 is a multifunctional protein involved in several critical functions like apoptosis, mitosis, hypoxia, inflammation and metabolic reprogramming [34,69]. The role of PKM2 in several cancers has been well-explored [70,73]. Furthermore, PKM2 participation in numerous cellular pathways, protein–protein interactions and nuclear transport indicates that it performs several non-glycolytic functions [32]. Perhaps PKM2 might also be involved directly or indirectly in regulating virus replication and pathogenesis through multiple pathways. Since the role of PKM2 in JEV pathogenesis has not been studied. The present study aims to understand the role of PKM2 in JEV replication. The results collectively show that PKM2 negatively regulates JEV replication.

We performed m.o.i. and time-dependent analysis of PKM2 expression in JEV-infected and uninfected Neuro-2a cells. The results revealed enhanced endogenous PKM2 expression in infected cells. Studies performed on other RNA viruses, such as classical swine fever virus (CSFV) and severe acute respiratory syndrome coronavirus 2, have also shown higher expression of PKM2 in infected cells [50,53]. To investigate the potential role of PKM2 in JEV replication, we conducted overexpression and knockdown experiments. We observed that JEV infection was significantly reduced in cells with overexpressed PKM2, while JEV replication was enhanced in cells with silenced PKM2. Similar results were observed upon treatment with metformin, which is a known inhibitor of PKM2 [74,75]. Altogether, these studies show that PKM2 plays a negative role in JEV replication.

PKM2 exists in two functional states: the active tetrameric state and the inactive dimeric state [76]. The affinity of dimeric PKM2 for PEP is low, contrary to that of tetrameric PKM2 [72]. PKM2 transforms into its dimeric form within cancer cells, enhancing glucose absorption and promoting the building up of glycolytic intermediates essential for anabolic activities, including the production of nucleic acids, amino acids and lipids [77,79]. Multiple molecules regulate the transition between the dimeric and tetrameric forms of PKM2. E7 oncoproteins, tyrosine kinase-mediated phosphorylation, acetylation and oxidation promote the development of the low-activity dimeric PKM2. Conversely, fructose-1,6-bisphosphate, serine and SAICAR facilitate the assembly of a highly active tetramer [80]. We investigated the expression level of different forms of PKM2 in JEV-infected neurons and observed an increase in all multimeric forms of PKM2 in JEV-infected cells relative to uninfected cells. It has been reported that tetrameric PKM2 mostly resides in the cytoplasm to perform glycolytic functions, whereas dimeric PKM2 frequently translocates to the nucleus to regulate gene transcription [81]. In JEV-infected neuronal cells, we observed higher PKM2 expression in both cytosolic and nuclear fractions compared to uninfected cells. This indicates the multifaceted role of all PKM2 isomers in JEV replication kinetics. It has been shown that dimeric nuclear PKM2 directly phosphorylates STAT3 at Tyr705, independent of the JAK2 and c-Src pathways, activating the transcription of MEK5 [82]. Tyr705 phosphorylation promotes the dimerization of STAT3, inducing its translocation to the nucleus and DNA binding [83]. Prior work has also shown that PKM2 interacts with STAT3, facilitating its activation by phosphorylating it at Tyr705, which subsequently promotes Th17 cell development [84]. Furthermore, it has been shown that the mutation of PKM2 at position Arg399 stabilizes its dimeric conformation, hence increasing its ability to phosphorylate STAT3 [82]. In the present study, we observed increased levels of both STAT3 and PKM2 in the nucleus of infected neuronal cells compared to uninfected ones. Furthermore, silencing of PKM2 reduced STAT3 dimerization and its translocation to the nucleus in infected cells compared to untreated infected cells. Altogether, these results indicated that PKM2 induces STAT3 activation to regulate JEV replication.

Several studies have linked PKM2 with the expression of proinflammatory cytokines [42,85,87]. Lipopolysaccharide has been shown to increase the PKM2 expression and STAT3 promoter binding, leading to TNF-*α* and IL-1*β* production in colorectal cancer [88]. Several proinflammatory cytokines are induced by neurotropic viruses and are responsible for neuronal death [89,92]. Consistent with the previous studies, we observed that neuronal cells infected with JEV resulted in enhanced proinflammatory cytokine expression during the early stages of infection. Moreover, overexpression of PKM2 enhanced the cellular antiviral response by inducing the expression of TNF-*α* and IL-1*β*, resulting in decreased viral multiplication and reduced release in the culture supernatant. Conversely, siRNA-mediated knockdown diminished the cellular antiviral response, facilitating JEV multiplication in neurons. This aligns with the previous research showing enhanced replication of JEV as a result of decreased levels of proinflammatory cytokines (IL-6 and TNF-*α*) in SOCS5-overexpressing microglial cells following JEV infection [93]. Taken together, we concluded that PKM2 negatively modulates JEV replication by inducing the expression of proinflammatory cytokines through STAT3 activation and its translocation to the nucleus.

PKM2 is a sticky protein and is known to interact with several host and viral proteins. It has been observed that PKM2 exhibits interactions with the pathogenic E7 protein of human papillomavirus and NS5B of the hepatitis C virus [31,54, 94]. The viral RNA polymerase is another target of PKM2 [95]. These interactions of PKM2 with viral proteins positively or negatively modulate virus infection. NS1 protein is a non-structural protein that plays an important role in viral infection and propagation by contributing to viral replication, virulence, immunological invasion and host complement system activation [96]. Multiple studies have shown that NS1 is a necessary cofactor in flavivirus RNA replication [97,100]. It has been discovered that NS1 in cells is found near vesicle packets and cytoplasmic vacuoles, where dengue virus replication takes place in Vero and C6/36 cells [97]. NS1, along with other transmembrane replicase components, plays a structural role in anchoring the replication complex to the membrane [15,101,104]. Experiments involving trans-complementation and mutagenesis in yellow fever virus or West Nile virus have demonstrated that whatever function NS1 plays, it occurs early in RNA replication [98,105]. Therefore, to elucidate the potential interaction between PKM2 and NS1, we performed *in silico* studies. The modelled mouse PKM2 and JEV NS1 protein structures were used for docking studies using ClusPro 2.0. The most stable PKM2-NS1 complex was found to have binding free energy of −150.85 kcal mol^−1^; the low binding energy shows a strong possible interaction. The analysis of the residues involved in the interaction showed the binding of PKM2 with the NS1 protein of JEV, involving several salt bridges and hydrogen bond linkages. We also analysed the rigidity and stability of proteins PKM2, NS1 and the PKM2-NS1 complex with the help of MD simulations, and comparisons were made using parameters such as RMSD, RMSF, Rg and SASA. The RMSD and RMSF patterns did not show any substantial shifts, which suggested the stability of PKM2, NS1 and PKM2-NS1 complex. Furthermore, the increase in Rg and SASA values after NS1 binding with PKM2 is because of the increase in the size of the complex compared to individual proteins. Also, the Rg and SASA patterns were overall consistent and suggested a stable complex. Microscopic studies also showed cellular co-localization of PKM2 and NS1 protein with a strong positive correlation between them, suggesting their probable interaction. Also, NS1 was found to localize in the ER of the infected cells with a strong positive correlation between NS1 and ER. Therefore, we concluded that PKM2 interacts with NS1 in the ER of infected cells. Furthermore, the interaction of PKM2 with NS1 was validated by the Co-IP assay. Since NS1 localizes in the ER of the infected cells, where it anchors the replication complex and facilitates replication [106,107]. Therefore, it can be suggested that both might co-localize in the ER of the infected cells, where PKM2 can interfere with the formation of the replication complex. Studies on the dengue virus and influenza A virus have shown that the NS1 protein is phosphorylated [108,109]. In the influenza virus, phosphorylation of NS1 at Thr49 has been shown to suppress its IFN antagonist activity, whereas phosphorylation at Ser205 was found to be essential for its polymerase-enhancing function [108,110]. Since dimeric PKM2 acts as a protein kinase, its interaction with NS1 could induce phosphorylation of NS1. However, depending on the position and type of phosphorylated residues, JEV replication can be regulated by PKM2. PKM2 also undergoes several post-translational modifications; one of them is *O*-GlcNAcylation, the attachment of *O*-linked *N*-acetylglucosamine to Ser and Thr residues [111,112]. *O*-GlcNAcylation of PKM2 at Thr405 and Ser406 promotes the Warburg effect by enhancing the translocation of PKM2 to the nucleus [112,113]. Several RNA viruses are known to induce the Warburg effect in infected cells, resulting in enhanced glycolysis [18,114, 115]. We have shown that Thr405 and Ser406 are also the interacting residues of PKM2 with NS1. This can affect the *O*-GlcNAcylation of PKM2, thereby regulating glycolysis and JEV replication in the infected cells. However, this requires experimental validation.

Although we have confirmed physical interaction between PKM2 and NS1 and shown the potential interacting residues, further site-directed mutagenesis of specific residues would help in identifying critical residues involved in the interaction and its effect on the overall binding affinity and function of the interacting proteins. Beyond its well-known function in metabolism, PKM2 also influences key cellular processes such as cell-cycle regulation, cell migration, apoptosis and autophagy [116,119]. Exploring PKM2 functions in the context of JEV neuropathogenesis could shed more light on the broader impact of PKM2 in JEV replication and uncover potential therapeutic targets. In addition, *in vivo* studies will help in better assessing the effect of PKM2 modulation on viral load, neuroinflammation and overall disease progression in a complex biological system. Overall, our work highlights PKM2 as a novel host factor that negatively regulates JEV replication in neurons by stimulating the expression of proinflammatory cytokines. Nonetheless, other host factors could also interact with PKM2 to modulate JEV infection. Further studies will help in understanding the crosstalk between PKM2 and JEV infection. The study paves a path to explore the possibility of using other host metabolic genes to elucidate the pathology of JEV in neurons.

## Supplementary material

10.1099/jgv.0.002140 Uncited Supplementary Material 1.

## Abbreviations

Co-IP: co-immunoprecipitation
2-DG: 2-deoxy-d-glucose
DMEM: Dulbecco's modified Eagle medium
DSS: disuccinimidyl suberate
ER: endoplasmic reticulum
GAPDH: glyceraldehyde-3-phosphate dehydrogenase
HIF-1*α*: hypoxia-inducible factor-1*α*
JEV: Japanese encephalitis virus
LDH: lactate dehydrogenase
MD: molecular dynamics
MM/GBSA: molecular mechanics/generalized born surface area
NS1: non-structural protein 1
PDB: Protein Data Bank
PEP: phosphoenol pyruvate
PKM2: pyruvate kinase muscle isozyme M2
PRRSV: porcine reproductive and respiratory syndrome virus
Rg: radius of gyration
RMSD: root mean square deviation
RMSF: root mean square fluctuation
SASA: solvent accessible surface area
STAT3: signal transducers and activators of transcription 3
