# Supplementary material for: Pyruvate kinase M2 modulates Japanese encephalitis virus replication in neuronal cells
Source: J Gen Virol. 2025 Sep 22;106(9):002140. doi: 10.1099/jgv.0.002140 (PMC12476147; doi:10.1099/jgv.0.002140)
Supplement: Uncited Supplementary Material 1. [file jgv-106-02140-s001.pdf]

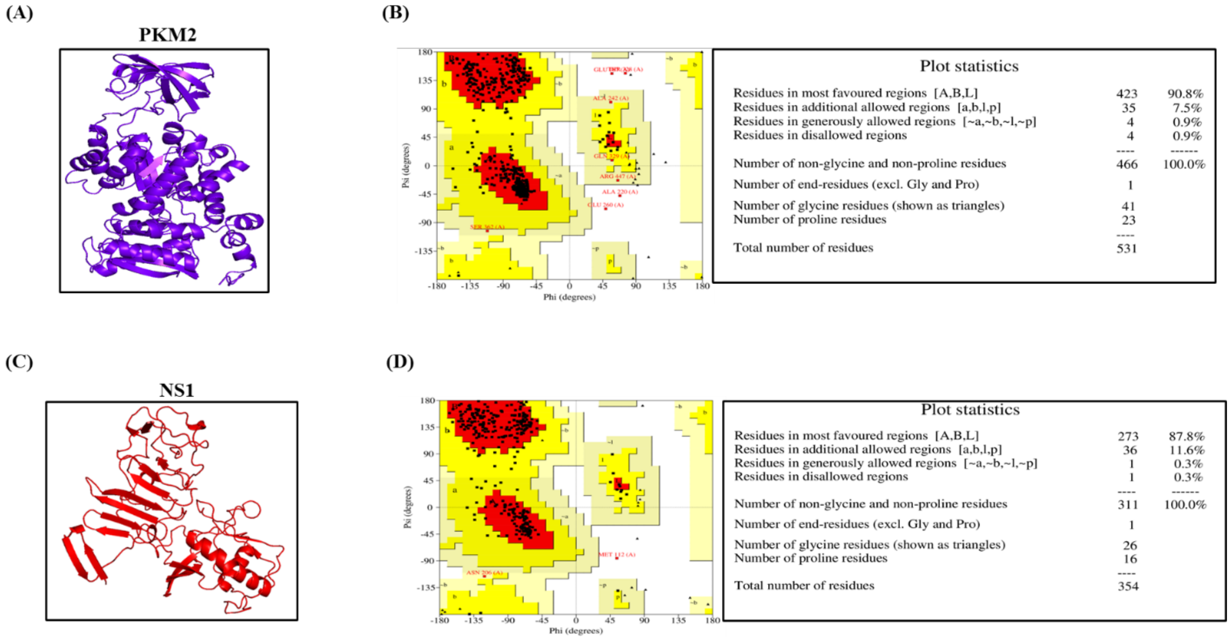

Figure S1. Structure modeling of JEV NS1 and mouse PKM2 protein. Figure depicting the three-dimensional model of mouse PKM2 protein (A). The modeled structure most similar to human PKM2 (PDB ID-3GR4) with RMSD value 0.74 was selected and analyzed for stereochemical stability using a Ramachandran plot, which showed 90.8% residues in most favoured regions, 7.5% in additional allowed regions, 0.9% in generously allowed regions, and only 0.9% in disallowed regions (B). Figure showing three-dimensional modeled structure of JEV NS1 protein (C). The predicted model was found to be most similar in structure to Zika virus NS1 (PDB ID-5K6K) with RMSD value 0.50, and was subsequently analyzed using a Ramachandran plot and showed 87.8% residues in most favoured regions, 11.6% in additional allowed regions, 0.3% in generously allowed regions, and only 0.3% in disallowed regions (D).

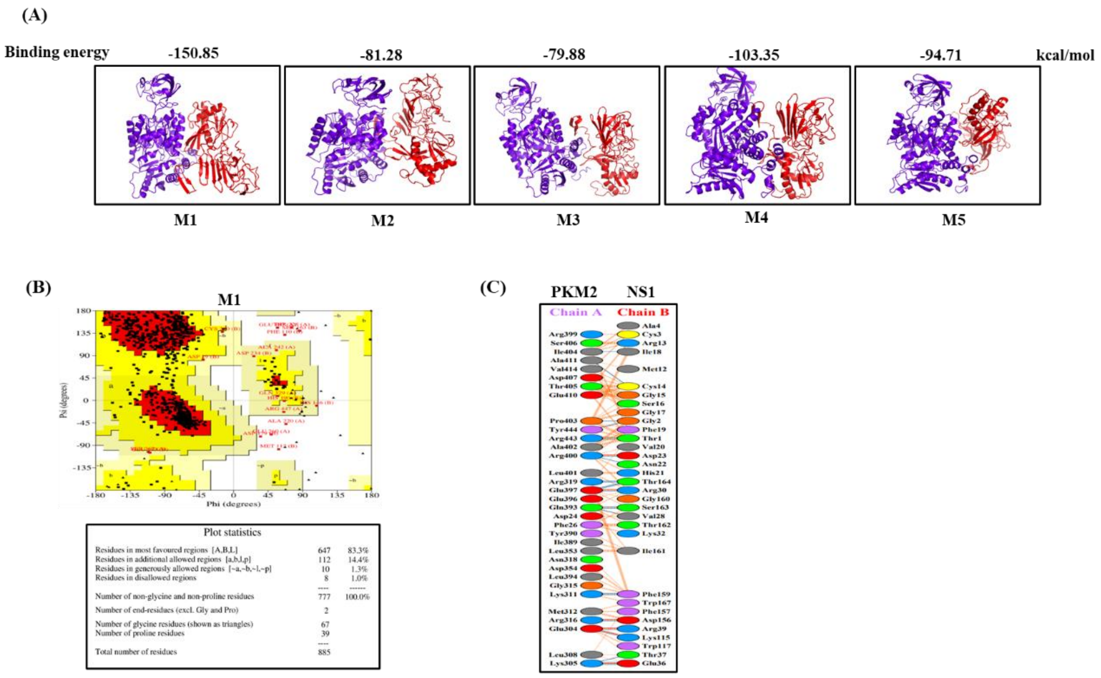

Figure S2. PKM2-NS1 Docking studies. Pictures of the predicted docking models (M1, M2, M3, M4 and M5) along with their binding free energies (A). Image representing Ramachandran plot of most stable PKM2-NS1 model based on binding free energy. There were 647 residues (83.3%) in the most favourable regions, 112 residues (14.4%) in additionally allowed regions, 10 residues (1.3%) in the generously allowed regions, and 8 residues (1.0%) in the disallowed regions (B). Diagrammatic representation of interacting amino acid residues involved in model M1 (C).

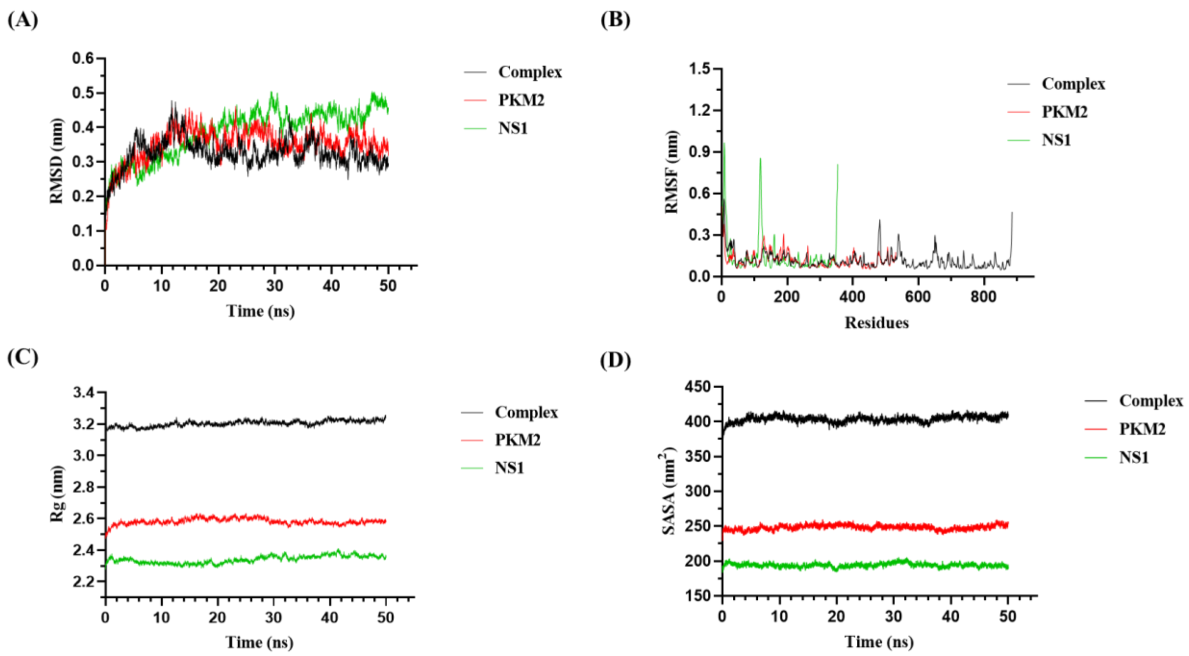

Figure S3. Molecular dynamics simulations. Graphs showing Root Mean Square Deviation (RMSD) analysis. The PKM2, NS1 and PKM2-NS1 complex achieved steadiness at 12 ns, 22 ns, and 17 ns, respectively and remained in equilibrium with only minor changes. The average RMSD values for PKM2, NS1 and PKM2-NS1 complex were 0.35 nm, 0.385 nm, and 0.325 nm, respectively (A). Graph representing Root Mean Square Fluctuations (RMSF) analysis. The PKM2, NS1 and PKM2-NS1 complex were stable throughout except for minor fluctuations around 110 residue region for NS1 and 500 residue region for PKM2 and PKM2-NS1 complex. The average RMSF values for PKM2, NS1 and PKM2-NS1 complex were 0.119 nm, 0.152 nm, and 0.154 nm, respectively (B). Radius of Gyration (Rg) analysis graph representing rigidity and compactness of the complex. Rg values for PKM2 acquired equilibrium at 10 ns and maintained it till the end, except for minor fluctuations around 20 ns and 30 ns, while NS1 achieved equilibrium at 6 ns and maintained it till the end, except for minor fluctuations at around 20-26 ns timeframe. The Rg values for the PKM2-NS1 complex showed minor fluctuations in the beginning. However, it achieved equilibrium at around 14 ns and maintained it till the end except for minor fluctuations at around 33–38 ns timeframe. The

average Rg values for PKM2, NS1, and PKM2-NS1 complex were 2.58 nm, 2.34 nm, and 3.20 nm, respectively (C). Graph showing Solvent Accessible Surface Area (SASA) analysis. The average SASA values obtained for the proteins PKM2, NS1, and PKM2-NS1 complex were 248.249 nm^2, 194.187 nm^2 and 403.759 nm^2, respectively (D).

**Interactions**	**PKM2 and NS1**
No. of interface residues	33:33:00
Interface area (A^2)	1711:1847
No. of salt bridges	6
No. of H-bonds	24
No. of non-bonded contacts	372

Table S1. The number of interface residues, interface area, salt bridges, H-bonds, and non-bonded contacts that exist between PKM2 and NS1 interaction.
